# Wave-controlled aliasing in parallel imaging magnetization-prepared gradient echo (wave-CAIPI MPRAGE) accelerates speed for pediatric brain MRI with comparable diagnostic performance

**DOI:** 10.1038/s41598-021-92759-y

**Published:** 2021-06-24

**Authors:** Younghee Yim, Mi Sun Chung, Su Yeong Kim, Na Mi Lee, Jun Soo Byun, Soo Ahn Chae

**Affiliations:** 1 Department of Radiology, Biomedical Research Institute, Chung-Ang University Hospital, Chung-Ang University College of Medicine, 102 Heukseok-ro, Dongjak-gu, Seoul, Republic of Korea; 2 Department of Pediatrics, Chung-Ang University Hospital, Chung-Ang University College of Medicine , 102 Heukseok-ro, Dongjak-gu, Seoul, Republic of Korea

**Keywords:** Medical research, Neurology

## Abstract

We aimed to compare accelerated post-contrast magnetization-prepared rapid gradient-echo (MPRAGE) using wave-controlled aliasing in parallel imaging (wave-CAIPI) with conventional MPRAGE as a reliable method to diagnose intracranial lesions in pediatric patients. A total of 23 consecutive pediatric patients who underwent post-contrast wave-CAIPI and conventional MPRAGE (scan time: 2 min 39 s vs. 5 min 46 s) were retrospectively evaluated. Two radiologists independently assessed each image for the presence of intracranial lesions. Quantitative [contrast-to-noise ratio (CNR), contrast rate (CR), and signal-to-noise ratio (SNR)] and qualitative parameters (overall image quality, gray-white matter differentiation, demarcation of basal ganglia and sulci, and motion artifacts) were also surveyed. Wave-CAIPI MPRAGE and conventional MPRAGE detected enhancing and non-enhancing intracranial lesions with 100% agreement. Although wave-CAIPI MPRAGE had a lower SNR (all *p* < 0.05) and overall image quality (overall analysis, *p* = 0.02) compared to conventional MPRAGE, other quantitative (CNR and CR) and qualitative parameters (gray-white differentiation, demarcation of basal ganglia and sulci, and motion artifacts) were comparable in the pooled analysis and between both observers (all *p* > 0.05). Wave-CAIPI MPRAGE was a reliable method for diagnosing intracranial lesions in pediatric patients as conventional MPRAGE at half the scan time.

## Introduction

In pediatric patients, magnetic resonance imaging (MRI) is an essential imaging modality that provides high soft tissue contrast and spatial resolution without exposing the patient to ionizing radiation^1, 2^. In particular, magnetization-prepared rapid acquisition gradient echo (MPRAGE) is one of the most commonly used sequence used to obtain high-resolution 3D T1-weighted images^3–5^. For the evaluation of congenital anomalies and variable-enhancing pathologies, such as metastasis or brain tumors, MPRAGE serves images with detailed anatomical structure with excellent gray-white differentiation^6, 7^. However, MPRAGE usually requires a long scan time to generate proper T1-weighted contrast using a long inversion time^3, 4^. Consequently, various parallel acquisition techniques are generally used for MPRAGE to reduce scan time^2^.

Unfortunately, the long scan time required for MRIs is a major obstacle for daily clinical use for pediatric patients^1, 2, 8–10^. This is particularly true with pediatric patients who frequently require sedation as their scans often have significant motion artifacts^2^. From this perspective, various efforts have been made to develop reliable fast sequences for pediatric brain MRIs^8, 9, 11–14^. Nevertheless, previous studies have been limited in terms of replacing MPRAGE. Fast MRIs with only T2-weighted images using single-shot fast spin-echo or half Fourier acquired single turbo spin echo have widely been used to evaluate hydrocephalus and shunt malformation; however, parenchymal abnormalities cannot be appropriately identified due to insufficient tissue contrast^8, 12^. Recently, a 2D image-based 1-min ultrafast brain MRI protocol has been suggested, however, 3D MRIs are essential in certain cases to access congenital anomalies or seizures in pediatric patients^14^. Therefore, further technical advances to increase the speed of MPRAGE are needed.

Wave-controlled aliasing in parallel imaging (CAIPI) is a cutting-edge parallel acquisition technique for obtaining high accelerated MRIs in clinical settings^15–17^. Wave-CAIPI was developed by combining bunched phase encoding and 2D-CAIPI to produce sinusoidal Gy and Gz gradients with a π/2 phase shift between the waveforms^16^. Consequently, it creates a characteristic corkscrew 3D k-space trajectory and disperses aliasing resulting from the parallel acquisition in all three spatial directions (x, y, z)^16^. Therefore, wave-CAIPI allows for highly accelerated images with low g-factor penalty and artifacts^16^. Recently, it has been suggested that, at half the scan time, pre-contrast wave-CAIPI MPRAGE may have sufficient spatial resolution for volumetric analysis in patients with dementia^17^. Therefore, we hypothesized that wave-CAIPI MPRAGE may also have sufficient spatial resolution for the diagnosis of intracranial lesions at a significantly reduced scan time in pediatric patients.

This study aimed to compare the diagnostic performance of post-contrast conventional 3D T1 weighted imaging MPRAGE and wave-CAIPI MPRAGE for intracranial lesions in pediatric patients. Additionally, quantitative and qualitative image parameters for both sequences were compared.

## Materials and methods

This retrospective study was approved by the institutional review board of Chung-Ang University Hospital (IRB number: 2007-034-19324), and informed consent was waived owing to the retrospective study design by the institutional review board of Chung-Ang University Hospital (IRB number: 2007-034-19324). Methods and results were reported following the STROBE (strengthening the reporting of observational studies in epidemiology) guidelines^18^.

### Study population

We retrospectively assessed consecutive patients who underwent post-contrast brain MRI examinations at a single tertiary center between September 2019 and March 2020. The inclusion criteria of this study were as follows: (a) patients with post-contrast brain MRI with both wave-CAIPI and conventional MPRAGE sequences, (b) age < 20 years, and (c) patients without any contraindication to MRI or contrast enhancement. The exclusion criterion was severe motion or metal artifacts. A total of 23 patients were finally enrolled in this study, and demographic and clinical data were retrospectively collected from the electronic medical records.

### Image acquisition

**Figure 1:**
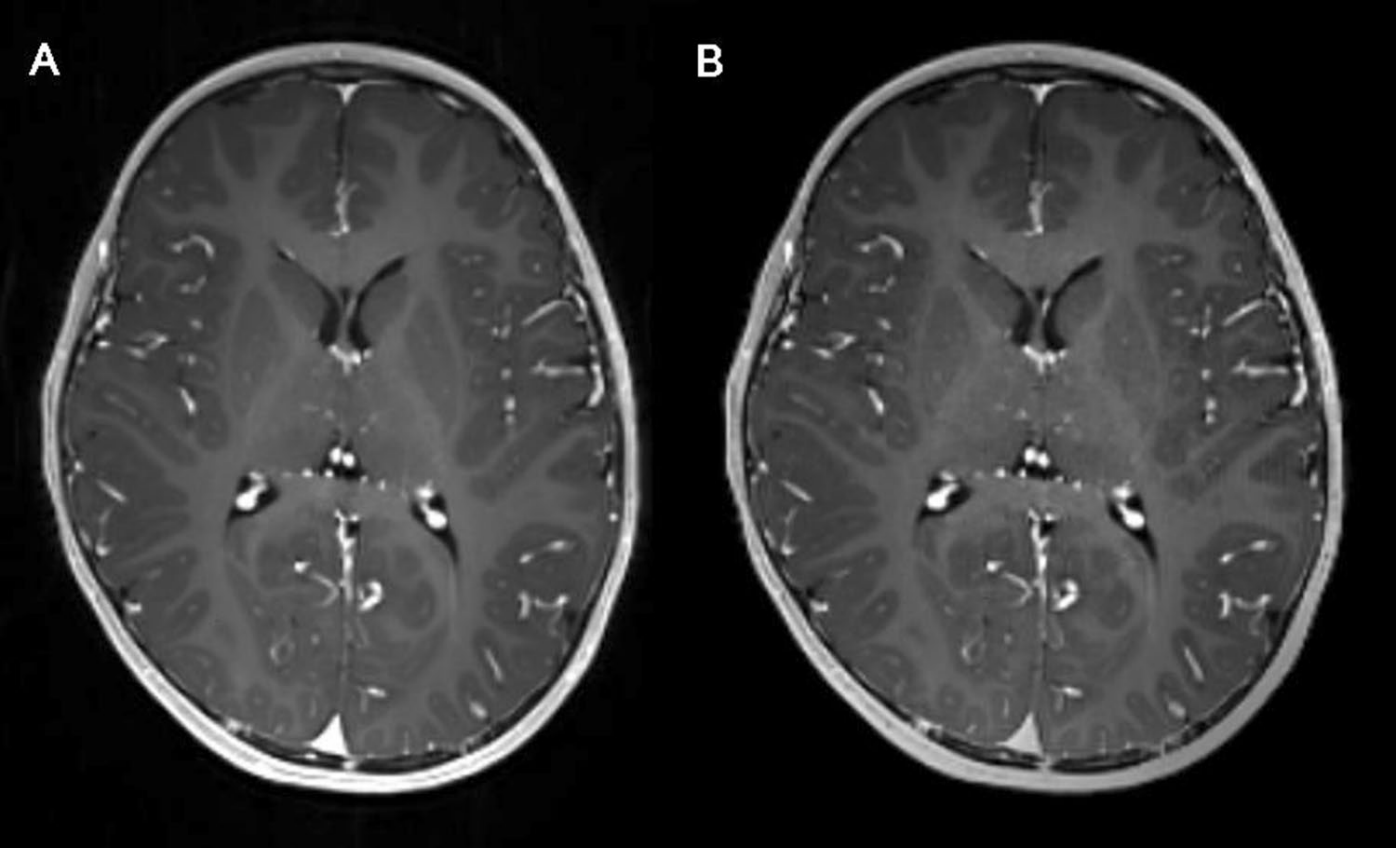
Conventional and wave-CAIPI MPRAGE. Representative image of conventional MPRAGE **(A)** and wave-CAIPI MPRAGE **(B)** of a boy aged 3 years and 7 months with seizures.

All MRI scans were performed using two 3 Tesla MRI systems (Magnetom Skyra, SIEMENS, Erlangen, Germany) with 64-channel head coils in the IDEA environment. Intravenous gadobutrol (Gadovist; Bayer Healthcare, Berlin, Germany; dose, 0.1 mL/kg) was administered using a 3-way stopcock. Post-contrast MR scanning were executed just after the injection of contrast media in the following order: conventional MPRAGE → wave-CAIPI MPRAGE (Fig. 1). Table 1 demonstrated detailed MRI parameters used for the post-contrast wave-CAIPI and conventional MPRAGE. The total acquisition times were 5 min 46 s for conventional MPRAGE and 2 min 39 s for wave-CAIPI MPRAGE.

**Table 1:** Image parameters.

	Conventional MPRAGE	Wave-CAIPI MPRAGE
Field of view (mm)	256 × 256	256 × 256
Voxel size (mm)	1 × 1 × 1	1 × 1 × 1
TR (ms)	2500	2500
TE (ms)	3.0	3.1
Flip angle	9.0	9.0
Band width (Hz)	240	240
TI (ms)	1100	1100
NEX	1	1
Parallel imaging method	GRAPPA	CAIPIRINHA
Acceleration factor (phase encoding direction)	2	2
Acceleration factor 3D (slice encoding direction)	–	2
Scan time	5 min 46 s	2 min 39 s

*CAIPIRINHA* controlled aliasing in parallel imaging results in higher acceleration, *GRAPPA* generalized autocalibrating partially parallel acquisitions, *MPRAGE* magnetization-prepared rapid gradient echo, *NEX* number of excitations, *TE* echo time, *TI* inversion time, *TR* repetition time, *wave-CAIPI* wave-controlled aliasing in parallel imaging.

### Image analysis

Two neuroradiologists (Y.Y. and M.S.C. with 9 and 10 years of experience, respectively) reviewed all images independently using the PACS system. They were blinded to the initial diagnosis, the sequence used, and the other observer’s results. Each observer assessed the images for each sequence separately with a 2-week interval to prevent recall bias.

To evaluate diagnostic performance, we determined whether enhancing and non-enhancing lesions were present and/or diagnosed in the brain parenchyma in both sequences. Pre-contrast MPRAGE, T2-weighted imaging, or FLAIR images were used to confirm the diagnosis in controversial cases. In patients with multiple pathologies, up to three different diagnoses were included for a single patient.

Quantitative analyses were performed by two neuroradiologists (Y.Y. and M.S.C.) using circular region of interest (ROI) measurements on axial images (Supplemental Fig. 1). For statistical analyses, the average values of both sides were considered the representative values. The ROIs were located in the gray and white matter of the frontal lobe at the level of centrum semiovale, both putamen, the frontal horn of both lateral ventricles, the central pons, and both cerebellums, avoiding artifacts or enhancing lesions such as vessels. Using the data from these ROIs, the following quantitative metrics were acquired: (1) contrast-to-noise ratio for white and gray matter (CNR_WM/GM_)^19, 20^, (2) contrast ratio for white matter and cerebrospinal fluid (CSF) (CR_WM/CSF_) and for gray matter and CSF (CR_GM/CSF_)^9, 19^, and (3) signal-to-noise ratio (SNR) at the level of the centrum semiovale, putamen, pons, and cerebellum. The CNR_WM/GM_ was defined as (signal intensity [SI] of white matter—SI of gray matter)/noise of white matter^19, 20^. We did not directly obtain the noise in the background because of the non-homogeneous noise distribution of the images with parallel acceleration^19, 21, 22^. Instead, we measured the standard deviation (SD) of the white matter. The contrast ratio for white matter and CSF (CR_WM/CSF_), and for gray matter and CSF (CR_GM/CSF_) were calculated as follows: CR_a/b_ = (SI_a_ – SI_b_)/(SI_a_ + SI_b_) × 100%^9, 19^. The SNR_a_ was defined as SI_a_/noise_a_^23^.

For the qualitative analysis, we assessed the following five image metrics: (1) overall image quality, (2) gray-white differentiation at the level of the lateral ventricle, (3) demarcation of the basal ganglia, (4) demarcation of sulci, and (5) motion artifacts. For overall image quality, gray-white differentiation, demarcation of basal ganglia and sulci, we visually analyzed whole images using 5-point Likert scales with the following criteria: 1 = non-diagnostic image quality; 2 = severe blurring resulting in significant limitation in evaluation; 3 = moderate blurring that slightly compromised evaluation; 4 = slight blurring that did not compromise image assessment; and 5 = excellent image quality. For the motion artifacts, we scored the whole image as follows: 1 = severe image artifacts; 2 = moderate artifacts; 3 = mild artifacts; 4 = minimal artifacts; and 5 = no artifacts.

### Statistical analysis

For statistical analysis, we used MedCalc Statistical Software version 19.6 (MedCalc Software Ltd, Ostend, Belgium). Percent agreement and the kappa (κ) value were used to assess the agreement between both conventional and wave-CAIPI MPRAGE for the presence of enhancing and non-enhancing lesions. The qualitative and quantitative image parameters were compared using the Wilcoxon signed-rank test. The strength of agreement using the κ values was categorized as follows: poor, < 0.20; fair, 0.21–0.40; moderate, 0.41–0.60; good, 0.61–0.80; and excellent, 0.81–1.00^24^. A *p*-value < 0.05 was considered statistically significant.

## Results

**Figure 2:**
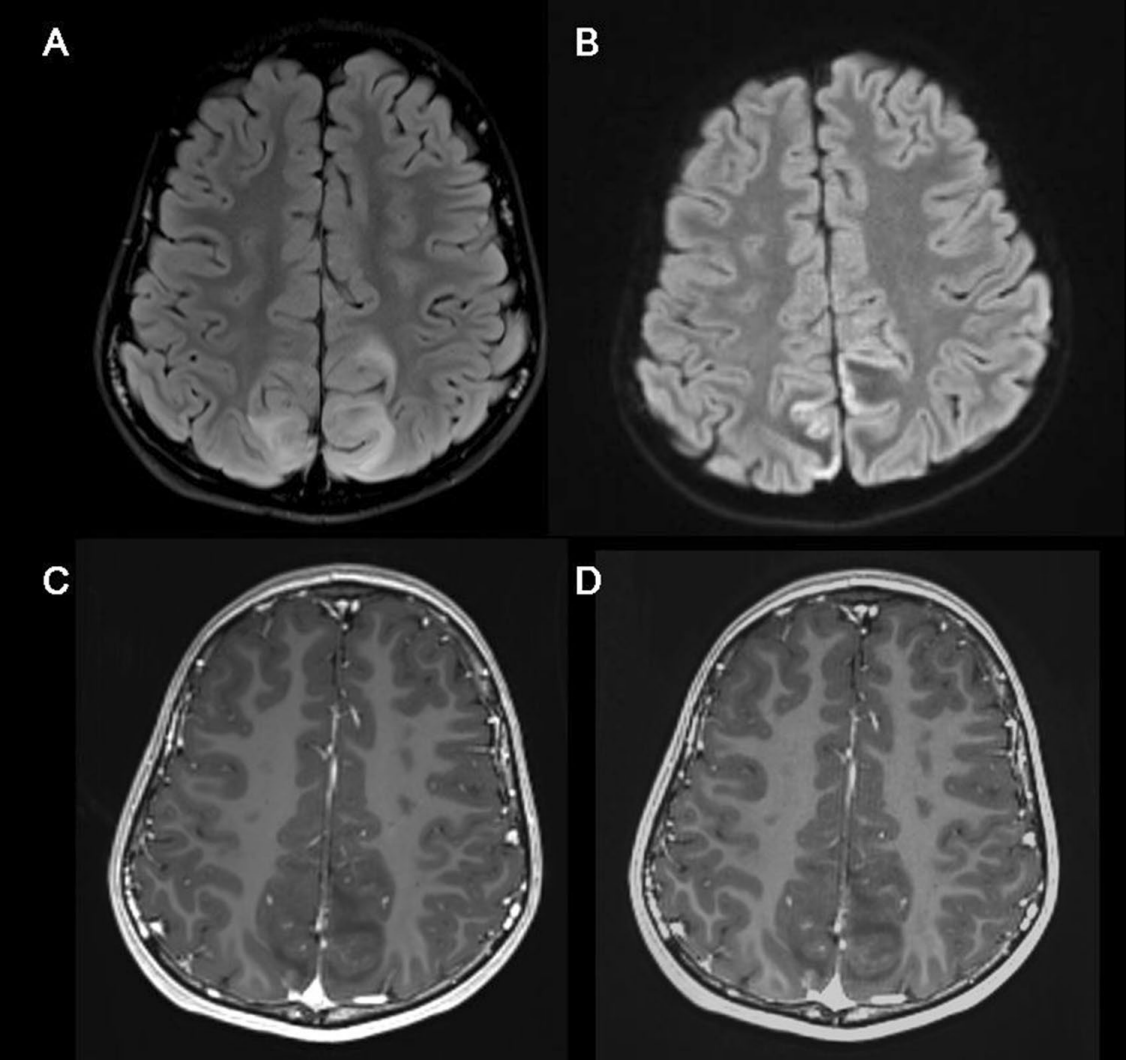
Probable encephalitis on conventional and wave-CAIPI MPRAGE. A boy aged 8 years and 2 months with convulsion-like movements and motor weakness of the lower limb, and fever shows confluent FLAIR high signal lesions in both parieto-occipital lobes **(A)** with some diffusion restriction in the cortexes **(B)**. After contrast injection, there is no evidence of enhancement on both conventional MPRAGE **(C)** and wave-CAIPI MPRAGE **(D)**. Based on the images and clinical and laboratory findings, the patient is diagnosed with encephalitis.

**Figure 3:**
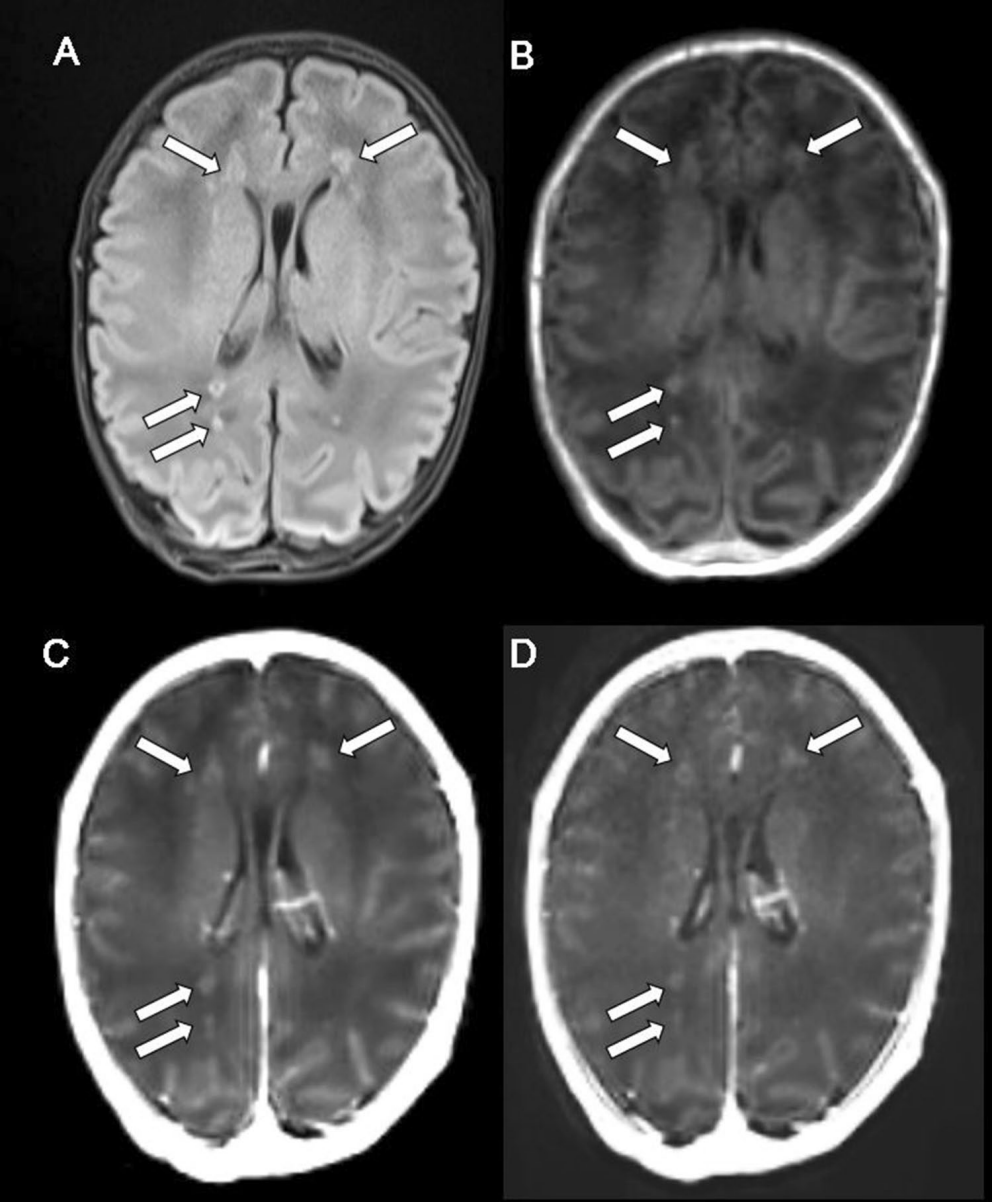
Periventricular leukomalacia on conventional and wave-CAIPI MPRAGE. A 1-month preterm neonate (gestational age: 36 weeks) undergoes an MRI for the evaluation of brain parenchymal lesions. Multifocal FLAIR **(A)** and precontrast T1 MPRAGE **(B)** high signal intensity lesions without enhancement on both conventional MPRAGE **(C)** and wave-CAIPI MPRAGE **(D)** which suggest areas of hemorrhage are discovered in both periventricular white matter. The patient is diagnosed with periventricular leukomalacia.

Detailed clinical information about the study population is summarized in Table 2. For detecting the presence of enhancing intracranial lesions, the agreement between the conventional and wave-CAIPI MPRAGE was 100%, with an excellent agreement (κ = 1.000) in both sequences in the pooled analysis (Table 2). Of the 23 patients, one patient had a probable developmental venous anomaly in the right frontal lobe. For detecting the presence or absence of non-enhancing lesions, the wave-CAIPI MPRAGE also was 100%, with an excellent agreement (κ = 1.000) (Table 2). The diagnosis of non-enhancing lesions included two congenital anomalies (migration anomaly including subependymal heterotopia and pachygyria, and corpus callosum agenesis), one arachnoid cyst, one old infarction, one old hemorrhage, one probable encephalitis (Fig. 2), one encephalomalacic change, and one periventricular leukomalacia (Fig. 3).

**Table 2:** Summary of study population.

#	Sex	Age	Clinical indication for brain MRI	Enhancing lesions (O1/O2)^a^		Non-enhancing lesions (O1/O2)^a^	
				Conventional MPRAGE	Wave-CAIPI MPRAGE	Conventional MPRAGE	Wave-CAIPI MPRAGE
1	M	8 yr 10 mo	Headache	−/−	−/−	−/−	−/−
2	M	8 yr 11 mo	Headache	−/−	−/−	−/−	−/−
3	M	11 yr 7 mo	Congenital anomaly	+ / +	+ / +	+ / +	+ / +
4	F	3 mo	Germinal matrix hemorrhage	−/−	−/−	+ / +	+ / +
5	M	6 yr 2 mo	Seizure	−/−	−/−	−/−	−/−
6	F	6 mo	Seizure	−/−	−/−	+ / +	+ / +
7	F	1 yr 1 mo	Seizure	−/−	−/−	−/−	−/−
8	F	1 mo	Seizure	−/−	−/−	−/−	−/−
9	M	2 mo	Congenital anomaly	−/−	−/−	−/−	−/−
10	F	9 yr 10 mo	Fever, vomiting	−/−	−/−	−/−	−/−
11	F	16 yr 6 mo	Headache, dizziness	−/−	−/−	−/−	−/−
12	M	15 yr 3 mo	Headache	−/−	−/−	−/−	−/−
13	M	1 yr 11 mo	History of hemorrhage	−/−	−/−	+ / +	+ / +
14	M	3 yr 7 mo	Seizure	−/−	−/−	−/−	−/−
15	M	8 yr 2 mo	Conversion, fever	−/−	−/−	+ / +	+ / +
16	F	9 yr 3 mo	Headache, vomiting	−/−	−/−	+ / +	+ / +
17	F	9 yr 6 mo	Seizure	−/−	−/−	−/−	−/−
18	F	12 yr 1 mo	Seizure	−/−	−/−	−/−	−/−
19	M	3 yr 1 mo	Seizure	−/−	−/−	−/−	−/−
20	M	1 mo	Congenital anomaly	−/−	−/−	+ / +	+ / +
21	F	4 yr 6 mo	Seizure	−/−	−/−	−/−	−/−
22	F	2 yr 2 mo	Seizure	−/−	−/−	−/−	−/−
23	F	4 yr 3 mo	Seizure	−/−	−/−	−/−	−/−

^a^O1/O2 indicates observer 1 / observer 2. Presence of lesion is indicated as + and absence of lesion is indicated as −.

For the quantitative image parameter analyses, the CNR_WM/GM_, CR_WM/CSF_, and CR_GM/CSF_ between the conventional and wave-CAIPI MPRAGE in the pooled analysis and between both observers did not differ significantly (*p* > 0.05 for all); Table 3). However, the SNR of the wave-CAIPI MPRAGE was lower than that of the conventional MPRAGE in the whole brain (*p* < 0.05 for all; Table 3).

**Table 3:** Quantitative image analysis.

	Conventional MPRAGE (median, interquartile range)	Wave-CAIPI MPRAGE (median, interquartile range)	P-value
**CNR**_**WM/GM**_			
Overall	5.08 (2.48–8.04)	4.31 (1.64 -6.87)	0.45
Observer 1	3.00 (1.27–5.60)	2.38 (1.44–4.20)	0.48
Observer 2	6.56 (3.50–10.31)	6.83 (4.35–10.29)	0.69
**CR**_**GM/CSF**_			
Overall	42.55 (41.30–44.79)	42.93 (41.39–46.15)	0.95
Observer 1	42.54 (40.17–45.59)	44.24 (41.31–47.91)	0.59
Observer 2	42.83 (39.17–45.45)	41.73 (39.27–46.56)	0.57
**CR**_**WM/CSF**_			
Overall	56.63 (54.31–58.12)	57.05 (55.63–58.50)	0.70
Observer 1	57.50 (52.78–59.10)	58.79 (55.17–61.02)	0.35
Observer 2	55.30 (47.54–58.46)	56.38 (51.50–57.95)	0.85
**SNR**_**centrum semiovale**_			
Overall	49.24 (37.56–61.04)	34.16 (23.10–42.57)	< 0.001
Observer 1	45.36 (37.91–50.46)	30.30 (20.78–38.65)	< 0.001
Observer 2	61.31 (35.96–73.93)	40.16 (24.85–50.35)	< 0.001
**SNR**_**putamen**_			
Overall	29.63 (23.18–39.73)	22.31 (17.49–25.76)	< 0.001
Observer 1	26.94 (23.30–30.03)	20.02 (14.18–21.41)	< 0.001
Observer 2	38.58 (25.99–51.86)	25.61 (23.70–32.05)	0.007
**SNR**_**pons**_			
Overall	20.99 (14.69–30.18)	17.67 (12.40–22.27)	0.038
Observer 1	20.64 (14.97–27.89)	15.62 (12.45–20.71)	0.08
Observer 2	21.34 (14.49–32.68)	18.75 (13.01–23.13)	0.24
**SNR**_**cerebellum**_			
Overall	37.43(28.94–53.94)	27.10 (18.91–32.44)	< 0.001
Observer 1	35.73 (23.43–43.01)	24.32 (16.61–28.27)	< 0.001
Observer 2	47.67 (35.15–58.62)	29.90 (20.27–37.67)	< 0.001

*CNR* contrast-to-noise ratio, *CR* contrast rate, *GM* gray matter, *MPRAGE* magnetization-prepared rapid gradient echo, *SNR* signal-to-noise ratio, *wave-CAIPI* wave-controlled aliasing in parallel imaging, *WM* white matter.

The qualitative analysis results demonstrated that the overall image quality of the wave-CAIPI MPRAGE was poorer than that of the conventional MPRAGE (*p* = 0.02 for the pooled analysis; Table 4). However, both sequences achieved a mean score > 4, suggesting good overall image quality. Moreover, other quantitative image parameters including gray-white differentiation, demarcation of basal ganglia, demarcation of sulci, and motion artifacts were not different between the two sequences (all *p* > 0.05; Table 4).

**Table 4:** Qualitative image analysis.

	Conventional MPRAGE [mean ± SD, median (interquartile range; range)]	Wave-CAIPI MPRAGE [mean ± SD, median (interquartile range; range)]	P-value
**Overall image quality**			
Overall	4.5 ± 0.7, 5 (4–5; 2–5)	4.5 ± 0.7, 4 (4–5; 3–5)	0.02
Observer 1	4.3 ± 0.7, 4 (4–5; 2–5)	4.0 ± 0.6, 5 (4–5; 3–5)	0.07
Observer 2	4.7 ± 0.7, 5 (5–5; 2–5)	4.2 ± 0.7, 4 (4–5; 3–5)	0.03
**Gray-white differentiation**			
Overall	5.0 ± 0.8, 5 (5–5; 1–5)	5.0 ± 0.6, 5 (5–5; 3–5)	0.91
Observer 1	4.7 ± 0.7, 5 (5–5; 2–5)	4.8 ± 0.5, 5 (5–5; 3–5)	1.00
Observer 2	4.7 ± 0.9, 5 (5–5; 1–5)	4.7 ± 0.6, 5 (5–5; 3–5)	0.88
**Demarcation of basal ganglia**			
Overall	5.0 ± 0.6, 5 (5–5; 2–5)	5.0 ± 0.3, 5 (5–5; 4–5)	1.00
Observer 1	4.9 ± 0.6, 5 (5–5; 2–5)	4.9 ± 0.3, 5 (5–5; 4–5)	1.00
Observer 2	4.9 ± 0.6, 5 (5–5; 2–5)	4.9 ± 0.3, 5 (5–5; 4–5)	1.00
**Demarcation of sulci**			
Overall	5.0 ± 0.8, 5 (5–5; 1–5)	5.0 ± 0.4, 5 (5–5; 4–5)	0.23
Observer 1	4.8 ± 0.8, 5 (5–5; 1–5)	4.7 ± 0.4, 5 (5–5; 4–5)	0.43
Observer 2	4.8 ± 0.8, 5 (5–5; 1–5)	4.8 ± 0.4, 5 (5–5; 4–5)	0.43
**Motion artifact**			
Overall	5.0 ± 0.6, 5 (5–5; 2–5)	5.0 ± 0.6, 5 (5–5; 3–5)	0.62
Observer 1	4.7 ± 0.5, 5 (5–5; 3–5)	4.7 ± 0.5, 5 (5–5; 3–5)	0.84
Observer 2	4.6 ± 0.7, 5 (5–5; 2–5)	4.6 ± 0.6, 5 (5–5; 3–5)	0.69

*MPRAGE* magnetization-prepared rapid gradient echo, *SD* standard deviation, *wave-CAIPI* wave-controlled aliasing in parallel imaging.

## Discussion

In the current study, wave-CAIPI MPRAGE showed perfect agreement with conventional MPRAGE for diagnosing intracranial lesions in pediatric patients with a 54% reduction in the acquisition time. Wave-CAIPI MPRAGE also had comparable CNR_WM/GM_, CR_WM/CSF_, and CR_GM/CSF_ to conventional MPRAGE. The SNR and overall image quality of wave-CAIPI MPRAGE were significantly poorer than those of conventional MPRAGE. However, the overall image quality of both wave-CAIPI MPRAGE and conventional MPRAGE had a median value of 4, suggesting good overall image quality with slight blurring that did not compromise image assessment. Moreover, other qualitative image metrics such as gray-white differentiation, demarcation of basal ganglia and sulci, and motion artifacts were also similar in both sequences. Considering the high diagnostic agreement and comparable image parameters, we concluded that post-contrast wave-CAIPI MPRAGE could be an alternative and faster acquisition method for brain MRI in pediatric patients in clinical practice.

Acquiring MRIs in pediatric patients is often difficult due to the degradation of image quality by unpredictable movement and the possible need for sedation^2^. The easiest and simplest way to decrease artifacts and sedative dose during MR scanning in pediatric patients is to reduce scan time^2, 8^. The key factor for saving scan time is the optimization of sequences by obtaining the necessary sequences as quickly as possible without compromising diagnostic information. Parallel imaging techniques also contributes two directions in pediatric imaging: to reduce scan time while retaining sensitivity or to increase sensitivity for tiny lesions instead of reducing scan time^2, 25–27^. However, conventional parallel techniques can be inadequate for fully managing the difficulties of MPRAGE scanning in pediatric patients, because MPRAGE is inevitably prolonged to obtain proper T1-weighted contrast and to apply long inversion time^2–4^. Of the pre-contrast MPRAGEs, wave-CAIPI MPRAGE has already been shown to have a considerably shorter scan time, with high scan-rescan reliability and high diagnostic agreement for visual and volumetric analysis in adult patients with dementia^17^. Compared with the scan time of other T1-weighted images such as 2D T1-weighted turbo spin echo images and 3D T1-weighted spin-echo images in our institution, wave-CAIPI MPRAGE also demonstrated significant scan time reduction in same field of view and slice thickness (3D T1-weighted spin-echo = 15 min 5 s and 3D T1-weighted spin-echo = 5 min 51 s). In the current study, we suggest that the wave-CAIPI technique could be an effective parallel acquisition method for MPRAGE in pediatric patients, based on the decreased scan time, high diagnostic performance, and comparable image metrics.

While various fast MRI techniques have been suggested for pediatric patients, they have disadvantages which include decreased sensitivity for small lesions, image blurring, and increased noise^2^. Like other sequences, wave-CAIPI MPRAGE also has drawbacks that need to be managed. Pre-contrast wave-CAIPI MPRAGE has been found to have more noise than conventional MPRAGE, particularly in the central brain, for the following reasons: (1) a relatively lower SNR is present in the central coil area compared with the periphery and (2) decreased SNR with the square root of the increase of acceleration factor^17, 26, 28^. Although technical progress has been made to minimize both noise amplification and wave-specific blurring artifacts, in this study, wave-CAIPI MPRAGE also had a lower SNR and decreased overall image quality than conventional MPRAGE. However, the mean value of the overall image quality was greater than 4, indicating slight blurring that did not compromise image assessment. In addition, wave-CAIPI MPRAGE achieved perfect diagnostic agreement and similar image parameters compared to conventional MPRAGE. Therefore, a decreased SNR and overall image quality in wave-CAIPI MPRAGE might have a negligible effect on radiologists’ assessments in clinical practice. Moreover, further improvements in postprocessing techniques, including de-noising and image regularization, could contribute to decreasing the level of noise in the wave-CAIPI MPRAGE images without excessive blurring.

With regard to motion artifacts, a previous technical study noted the possibility of more severe motion artifacts using the wave-CAIPI technique^15, 16^. In contrast, previous clinical studies using similar wave-CAIPI susceptibility-weighted imaging and pre-contrast wave-CAIPI MPRAGE reported fewer or a similar amount of motion artifacts in wave-CAIPI accelerated images compared to conventional images^17, 29, 30^. In line with these results, the motion artifacts noted in this study were similar to those of conventional MPRAGE. As the acquisition time wave-CAIPI MPRAGE is shorter, there is less likelihood of creating motion artifacts due to patient motion during MRI scanning. This result is promising for the application of wave-CAIPI MPRAGE in motion-prone pediatric patients. However, further studies with larger study populations are needed to confirm these findings regarding wave-CAIPI MPRAGE in pediatric patients.

This study has several limitations. First, a small study population from a single third referral center was used for this retrospective study and a small number of patients with parenchymal lesions was included in this study. Therefore, an unavoidable selection bias could have been introduced and there is a limitation to specify preferred clinical indication of each sequences based on the results of this study. Second, we could not randomize the order of the conventional and wave-CAIPI MPRAGE scans because of the retrospective nature of this study and fixed scan order could introduce bias in this study. Previous studies reported improved detectability of intracranial lesions in later sequences with prolonged scan delay, therefore CNR and CR of wave-CAIPI could be overestimated^31, 32^. In addition, motion artifact of wave-CAIPI MPRAGE might be underestimated. Third, complete blindness of visual analysis to the sequence type may not possible because expert readers could recognize the distinguishing features of the various sequences. Consequently, the reader blinding could be compromised and the selection bias might occur. We try to minimize this bias by simultaneously providing objective results including quantitative analysis. Fourth, we calculated SNR using AAPM/ACR method. Previously AAPM/ACR method had been reported to have limitation in SNR measurement for images using parallel acquisition due to high variability compared to the other methods including NEMA method^33^. However, the other suggested methods use complex methods for the SNR calculation and it was technically impossible in our clinical setting. Lastly, the diagnoses were based on MRI findings because pathological confirmations are difficult with intracranial lesions. Thus, we designed this study to survey diagnostic agreement and to compare various image parameters rather than for diagnostic accuracy based on pathologic confirmation. Based on our results, further studies regarding variable clinical applications and the optimization of scan parameters for wave-CAIPI MPRAGE in pediatric patients are warranted.

In conclusion, wave-CAIPI MPRAGE was found to be comparable to conventional MPRAGE for diagnosing intracranial lesions in pediatric patients, at half the scan time. Moreover, wave-CAIPI MPRAGE had comparable qualitative parameters regarding anatomical details and motion artifacts, and quantitative parameters, including CNR and CR, compared to conventional MPRAGE. Considering the decreased scan time and similar diagnostic performance, wave-CAIPI MPRAGE could be a potential alternative method for obtaining pediatric brain MRIs in daily clinical practice.

## Supplementary Information

Supplementary Information.
