# Supplementary material for: Wave-controlled aliasing in parallel imaging magnetization-prepared gradient echo (wave-CAIPI MPRAGE) accelerates speed for pediatric brain MRI with comparable diagnostic performance
Source: Sci Rep. 2021 Jun 24;11:13296. doi: 10.1038/s41598-021-92759-y (PMC8225910; doi:10.1038/s41598-021-92759-y)
Supplement: Supplementary file 1 — Supplementary Information. [file 41598_2021_92759_MOESM1_ESM.pdf]

Wave-controlled aliasing in parallel imaging magnetization-prepared gradient echo (Wave-CAIPI MPRAGE) accelerates speed for pediatric brain MRI with comparable diagnostic performance

Manuscript category: Original Research

Younghee Yim, MD, PhD[1;] Mi Sun Chung, MD, PhD[1*]; Su Yeong Kim, MD [2]; Na Mi Lee, MD, PhD [2]; Jun Soo Byun, MD, PhD[1]; Soo Ahn Chae, MD, PhD [2]

[1]Department of Radiology, Biomedical Research Institute, Chung-Ang University Hospital, Chung-Ang University College of Medicine, 102 Heukseok-ro, Dongjak-gu, Seoul, Republic of Korea

[2]Department of Pediatrics, Chung-Ang University Hospital, Seoul 06973, South Korea

College of Medicine, Chung-Ang University, Seoul 06911, South Korea

Supplemental Materials

Supplemental Figure 1. The location of region of interest for quantitative analysis

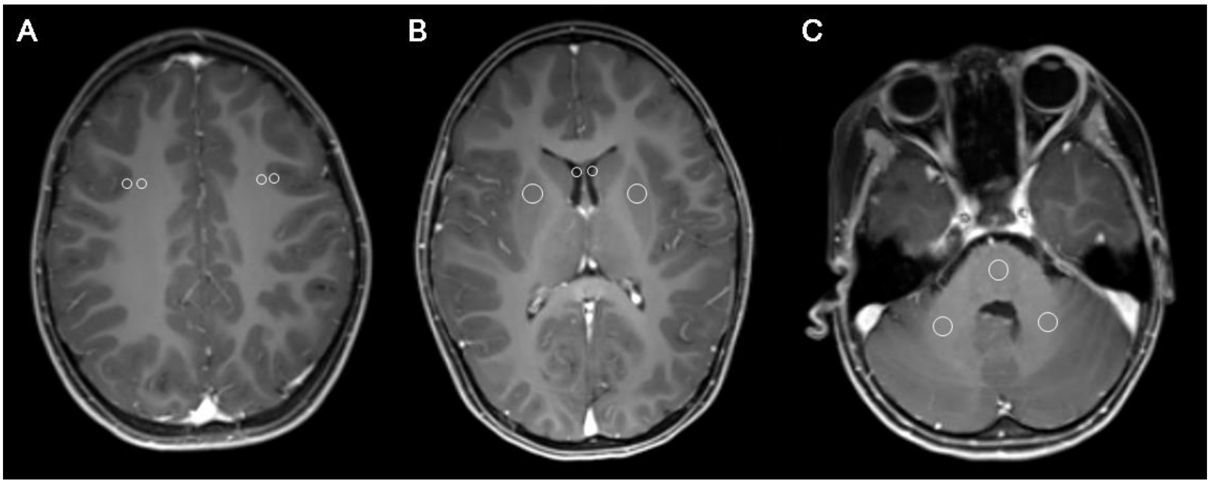

Quantitative analyses were performed by two neuroradiologists (Y.Y. and M.S.C.) using circular region of interest (ROI) measurements on axial images. The locations of ROIs were as follows; 1) the gray and adjacent subcortical white matter of the frontal lobe at the level of centrum semiovale of both sides (ROI size: 5 - 8 mm^2, 1A), 2) both putamen (ROI size: 20-30 mm^2, 1B) and the frontal horn of both lateral ventricles (ROI size: 5 - 8 mm^2, 1B) at the level of anterior corpus callosum, and 3) the central pons and both cerebellums and the level of middle cerebellar peduncle (ROI size: 20-30 mm^2, 1C). The measurements of ROI were performed with avoidance of artifacts or enhancing lesions such as vessels.

Supplemental Table 1. Raw data of qualitative analysis

#	Overall image quality				Gray-white differentiation				Demarcation of basal ganglia				Demarcation of sulci				Motion artifact			
	Conventional MPRAGE		Wave-CAIPI MPRAGE		Conventional MPRAGE		Wave-CAIPI MPRAGE		Conventional MPRAGE		Wave-CAIPI MPRAGE		Conventional MPRAGE		Wave-CAIPI MPRAGE		Conventional MPRAGE		Wave-CAIPI MPRAGE	
	O1	O2	O1	O2	O1	O2	O1	O2	O1	O2	O1	O2	O1	O2	O1	O2	O1	O2	O1	O2
1	4	5	4	5	5	5	5	5	5	5	5	5	5	5	5	5	5	5	5	5
2	4	4	3	4	5	5	5	5	5	5	5	5	5	5	5	5	5	4	5	4
3	5	5	3	3	5	5	4	4	5	5	5	5	5	5	4	4	5	5	4	4
4	5	4	5	4	5	4	5	4	5	5	5	5	4	5	4	5	5	4	5	4
5	4	5	4	5	5	5	5	5	5	5	5	5	5	5	5	5	5	5	5	4
6	5	5	3	3	4	4	4	3	5	5	4	4	5	5	4	4	5	5	4	3
7	5	5	4	4	5	5	5	5	5	5	5	5	5	5	5	5	5	5	5	5
8	2	2	4	5	2	1	5	5	2	2	5	5	1	1	5	5	3	2	5	5
9	4	4	3	3	4	4	3	3	5	5	5	5	5	5	4	4	4	4	3	3
10	4	5	4	5	5	5	5	5	5	5	5	5	5	5	5	5	4	4	5	5
11	4	5	5	5	5	5	5	5	5	5	5	5	5	5	5	5	5	5	5	5
12	4	5	4	4	5	5	5	5	5	5	5	5	5	5	5	5	5	5	5	5
13	4	5	4	4	5	5	4	5	5	5	4	4	5	5	4	4	5	5	4	5
14	5	5	4	4	5	5	5	5	5	5	5	5	5	5	5	5	5	5	5	5

15	5	5	4	4	5	5	5	5	5	5	5	5	5	5	5	5	5	5	5	5
16	5	5	5	4	5	5	5	5	5	5	5	5	5	5	5	5	5	5	5	5
17	3	5	4	3	4	5	5	5	5	5	5	5	5	5	4	4	4	4	4	4
18	4	5	4	4	5	5	5	5	5	5	5	5	5	5	5	5	4	5	4	5
19	4	5	4	5	5	5	5	5	5	5	5	5	5	5	5	5	5	4	5	4
20	5	4	5	4	5	5	5	5	5	5	5	5	5	5	5	5	5	5	5	5
21	5	5	4	5	5	5	5	5	5	5	5	5	5	5	5	5	5	5	5	5
22	5	5	5	5	5	5	5	5	5	5	5	5	5	5	5	5	5	5	5	5
23	4	5	4	5	5	5	5	5	5	5	5	5	5	5	5	5	5	5	5	5
